# Dermoid Cyst With Adnexal Torsion Presenting as Acute Appendicitis in a Young Premenstrual Patient

**DOI:** 10.7759/cureus.101494

**Published:** 2026-01-13

**Authors:** Ioannis Korkontzelos, Georgios Athanasiou, George Mpourazanis, Konstantinos Lantavos, Panagiotis Tsirkas, Savvas Tsigas, Dimitrios Alefragkis, Pinelopi Kitsakou, Eufemia Balassi, Demosthenes Ziogas

**Affiliations:** 1 Department of Obstetrics and Gynecology, Ioannina General Hospital "G. Chatzikosta", Ioannina, GRC; 2 Department of Surgery, Ioannina General Hospital "G. Chatzikosta", Ioannina, GRC; 3 Department of Radiology, Ioannina General Hospital "G. Chatzikosta", Ioannina, GRC; 4 Department of Nursing, School of Health Sciences, National and Kapodistrian University of Athens, Athens, GRC; 5 Department of Anaesthesiology, Ioannina General Hospital "G. Chatzikosta", Ioannina, GRC; 6 Department of Pathology, Ioannina General Hospital "G. Chatzikosta", Ioannina, GRC

**Keywords:** acute abdomen, adnexal torsion, appendicitis, dermoid cyst, surgery

## Abstract

Adnexal masses are relatively frequent in adults but also present in prepubertal females. Mature ovarian teratomas, also called dermoid cysts, are slowly growing disorders that could result in adnexal torsion (AT), rupture, or hemorrhage. In these cases, low abdominal pain or acute abdomen are the commonest symptoms, leading the patient to the medical emergency unit. In adolescence, early diagnosis of this pathology is not uncommon since it can be disclosed in a routine gynecological examination. Unfortunately, in young premenarchal females, routine examination or a screening program for dermoid cysts does not exist. In cases of abdominal pain due to AT, early attendance to the hospital, prompt diagnosis, and timely surgical intervention are of paramount importance, increasing the possibility of minimal surgery and preservation of the ovary. Concomitant inflammation of the neighboring organs, such as the appendix, should also be considered.

## Introduction

Ovarian dermoid cysts (ODCs), also known as ovarian mature cystic teratomas, derive from germ cells (three layers: ectoderm, mesoderm, and endoderm). ODCs are considered neoplasms mainly benign, consisting approximately 20% of tumors in the female population. ODCs represent approximately 70% of all benign neoplasms of the ovary under the age of 30, while malignant transformation is reported rarely (1-3%) [1,2].

Adnexal torsion (AT) consists of the partial or complete twisting of the ovarian pedicle around its axis, including also the salpinx, with consequent impairment of the blood flow and final necrosis. Instead, ovarian torsion solely is the twisting of the ovarian pedicle around the mesovarium. It is considered a major cause of acute abdomen presenting in adolescence, but also during the neonatal period. The incidence of ovarian torsion is reported between 4.9/100.000 and 20-30/100.000 in the female population, with a mean age of 13 up to 14.5 years, but younger average ages have also stated [3,4].

The clinical manifestation of AT is, most of the time, obscure, and delayed diagnosis or misdiagnosis is not uncommon. In cases of right-sided ovarian torsion in a premenarchal child, the patient could present with symptoms mimicking acute appendicitis. A single-center review study reported that, in 38% of premenarchal females, the delay in diagnosis ranged from 12 hours up to four days and the average time between torsion and surgery ranged from 16 to 100 hours [5]. Another study reported that 51% of these young patients presented with intermittent, non-severe pain, and, in 47%, the pain was present for 48 hours or more [6]. Prieto et al. considered that young premenarchal females have an increased risk of torsion even without an adnexal mass and a higher risk of necrosis and noted that 7-10 hours of pain are enough to establish tissue necrosis. Ιn 34% of premenarchal and in 17% of postmenarchal girls, these necrotic lesions have already established at the time of the operation [5].

## Case presentation

This case involves an eight-year-old girl who was referred to the outpatient clinic of our hospital with the initial diagnosis of acute appendicitis. The medical history of the young patient was insignificant. The pain had a gradual onset, starting 48 hours before and worsening over the last few hours. Clinical examination revealed acute abdominal pain, mainly in the right lower abdomen, with rebound tenderness and positive McBurney’s and Rovsing’s signs. Four to five hours before nausea was also established. Body temperature was 37°C. Laboratory investigation revealed leukocytosis with elevated neutrophils, elevated C-reactive protein (CRP), and alkaline phosphatase (ALP), with the rest of the examination being normal (Table 1).

**Table 1:** Laboratory examinations on admission, during hospitalization and in the first and second year follow-up. Abbreviations: Ht = hematocrit; Hb = hemoglobin; WBC = white blood cell count; NEUT = neutrophils; LYMPH = lymphocytes; CRP = C-reactive protein; INR = international normalized ratio; AST = aspartate aminotransferase; ALT = alanine aminotransferase; ALP = alkaline phosphatase; γGT = gamma-glutamyl transferase; AMY = amylase; UA = uric acid; LDH = lactate dehydrogenase; GLU = glucose; UREA = blood urea nitrogen; CREA = creatinine; K⁺ = potassium; Na⁺ = sodium; Mg²⁺ = magnesium; Cl⁻ = chloride; Ca²⁺ = calcium; PO₄³⁻ = phosphate; CA125 = cancer antigen 125; CA19-9 = carbohydrate antigen 19-9; CA15-3 = cancer antigen 15-3; CEA = carcinoembryonic antigen; β-HCG = beta-human chorionic gonadotropin; FSH = follicle-stimulating hormone; LH = luteinizing hormone; E2 = estradiol; PRG = progesterone

Parameter	Day 0 (Admission and operation)	Day 1	Day 4 (exit)	Follow up (1 year)	Follow up (2 years)	Reference range
Ht	41%	37%	35%	37%	38%	36-52%
Hb	14.8 g/dL	12.9 g/dL	11.5 g/dL	12.7 g/dL	13 g/dL	11.8-17.8
WBC	15.4 k/μL	14.03 k/μL	11.20 k/μL	13 k/μL	12.1 k/μL	4-11 k/μL
NEUT	94%	77.9%	75%	76%	74%	40-75%
LYMPH	51%	14.3%	10%	13%	11%	20-45%
CRP	2mg/dL	1.7 mg/dL	0.80 mg/dL	0.85 mg/dL	0.90 mg/dL	0-0.80 mg/dL
INR	1.04	1.0	-	0.94	0.96	1-1.13
AST	29 U/L	26 U/L	22 U/L	24 U/L	28 U/L	5-33 U/L
ALT	23 U/L	22 U/L	20 U/L	27 U/L	26 U/L	5-32 U/L
ALP	274 IU/L	205 IU/L	180 IU/L	193 IU/L	201 IU/L	13-125 IU/L
γGT	15 IU/L	13 IU/L	12 IU/L	8 IU/L	11 IU/L	5-31 IU/L
AMY	29 IU/L	30 IU/L	28 IU/L	29 IU/L	31 IU/L	28-100 IU/L
UA	3 mg/dL	3.5 mg/dL	2.8 mg/dL	4.1 mg/dL	2.9 mg/dL	2.3-6.1 mg/dL
LDH	216 IU/L	199 IU/L	185 IU/L	131 IU/L	168 IU/L	120-230 IU/L
GLU	133 mg/dL	92 mg/dL	85 mg/dL	78 mg/dL	83 mg/dL	70-115 mg/dL
UREA	38 mg/dL	26 mg/dL	24 mg/dL	29 mg/dL	22 mg/dL	10-50 mg/dL
CREA	0.59 mg/dL	0.56 mg/dL	0.64 mg/dL	0.60 mg/dL	0.62 mg/dL	0.5-1.1 mg/dL
K+	3.6 mmol/L	4.4 mmol/L	3.5 mmol/L	4.1 mmol/L	3.9 mmol/L	3.5-5.1 mmol/L
Na+	138 mmol/L	137 mmol/L	135 mmol/L	140 mmol/L	145 mmol/L	136-146 mmol/L
Mg++	1.68 mEq/L	1.76 mEq/L	1.40 mEq/L	1.88 mEq/L	1.92 mEq/L	1.3-2.1 mEq/L
Cl	101 mmol/L	100 mmol/L	99 mmol/L	101 mmol/L	98 mmol/L	98-106 mmol/L
Ca++	10.3 mg/dL	9.3 mg/dL	9 mg/dL	9.7 mg/dL	1 mg/dL	8.2-10.5 mg/dL
PO4--	3.5 mg/dL	3.9 mg/dL	4.3 mg/dL	4.8 mg/dL	5.2 mg/dL	4-7 mg/dL
CA125	20 U/L	-	-	-	-	< 35 U/L
CA19-9	20.4 U/L	-	-	-	-	< 37 U/L
CA15-3	18 U/L	-	-	-	-	< 31.3 U/L
CEA	1 ng/gL		-	-	-	< 5 ng.gL
β-HCG	-	-	-	-	-	0-5 mIU/mL
FSH	-	-	-	2.7 mlU/mL	3.0 mlU/mL	0.4-4.2 mlU/mL
LH	-	-	-	0.10 mlU/mL	0.12 mlU/mL	<0.3 mlU/mL
E2	-	-	-	4 pg/mL	24.0 pg/mL	< 48 pg/mL
PRG	-	-	-	1.30 nmol/L	0.51 nmol/L	0.4-270 nmol/L

**Figure 1:**
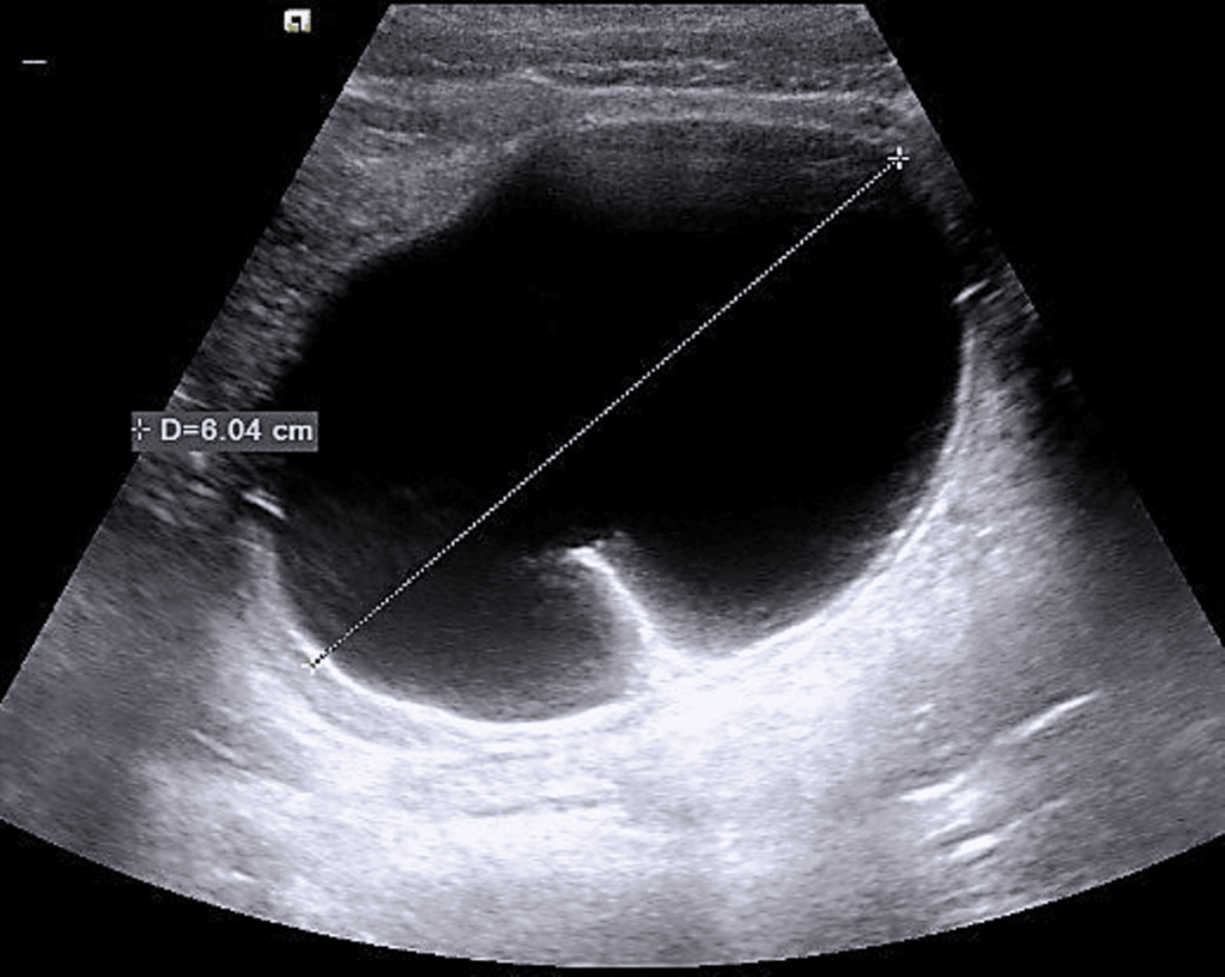
Abdominal ultrasound revealing the right-side ovarian cyst measuring 6 cm.

**Figure 2:**
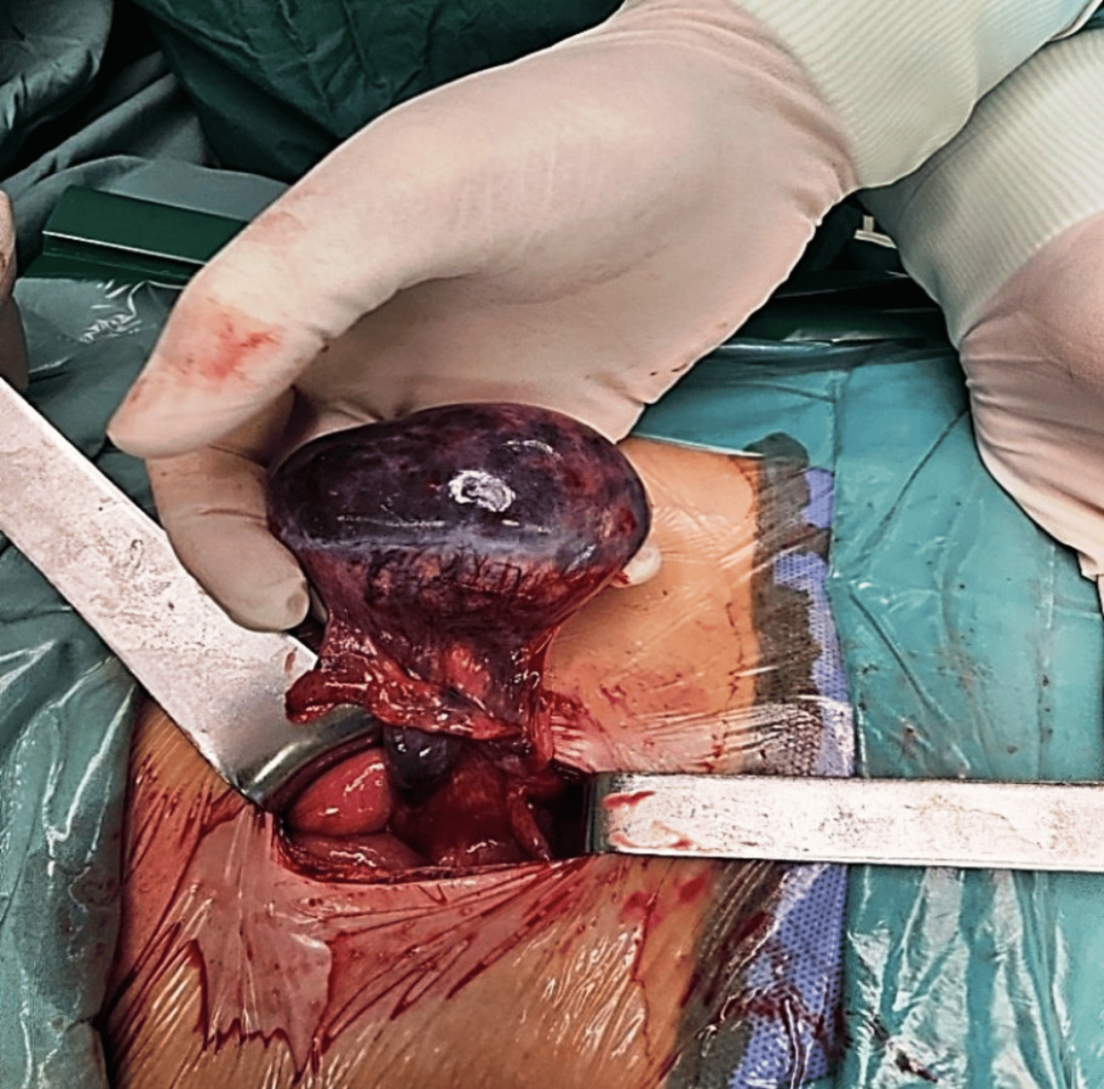
Adnexal torsion through McBurney’s incision with obvious necrosis.

**Figure 3:**
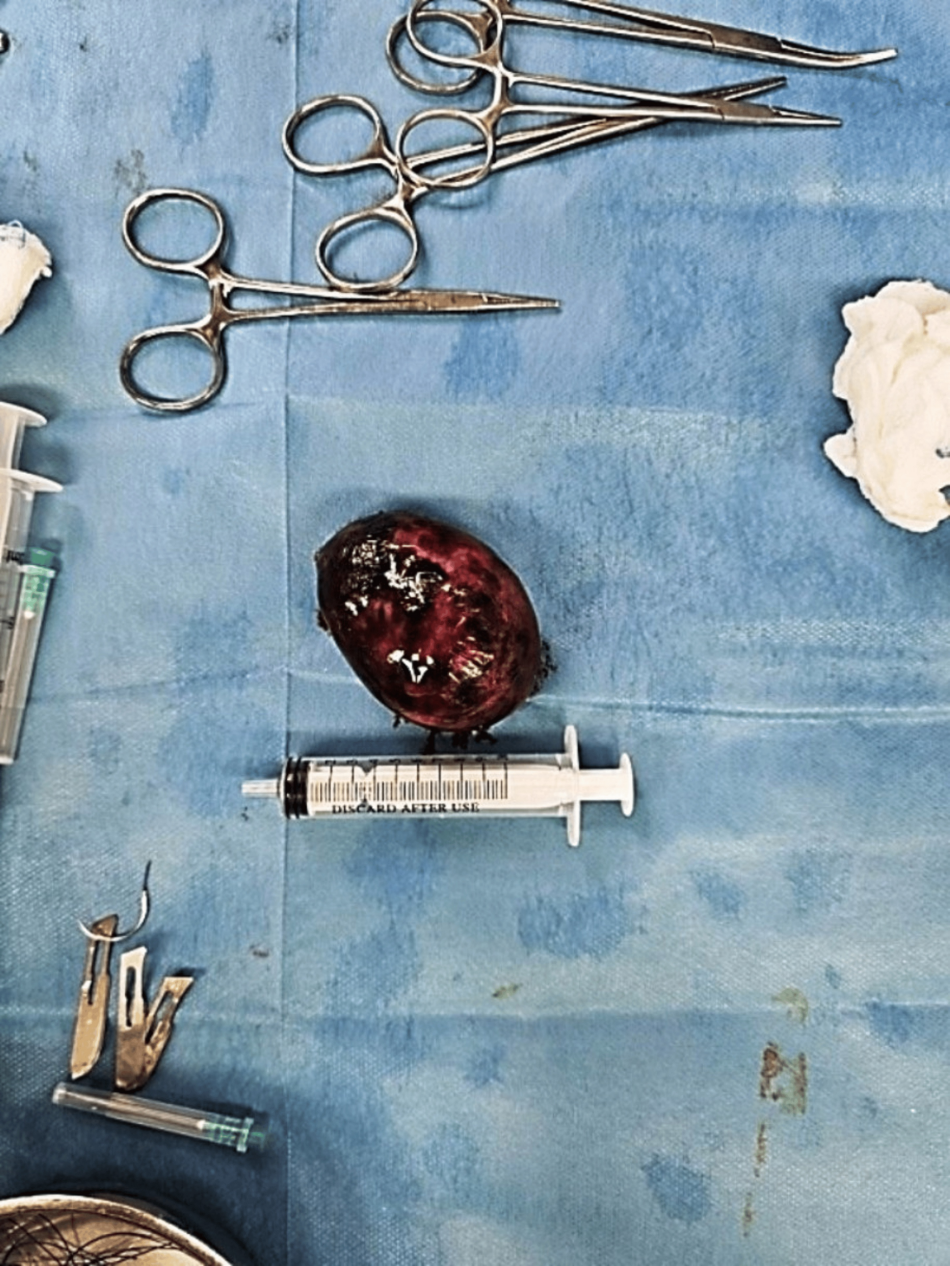
Macroscopic appearance of the excised specimen after salpingoophorectomy measuring 10 cm.

**Figure 4:**
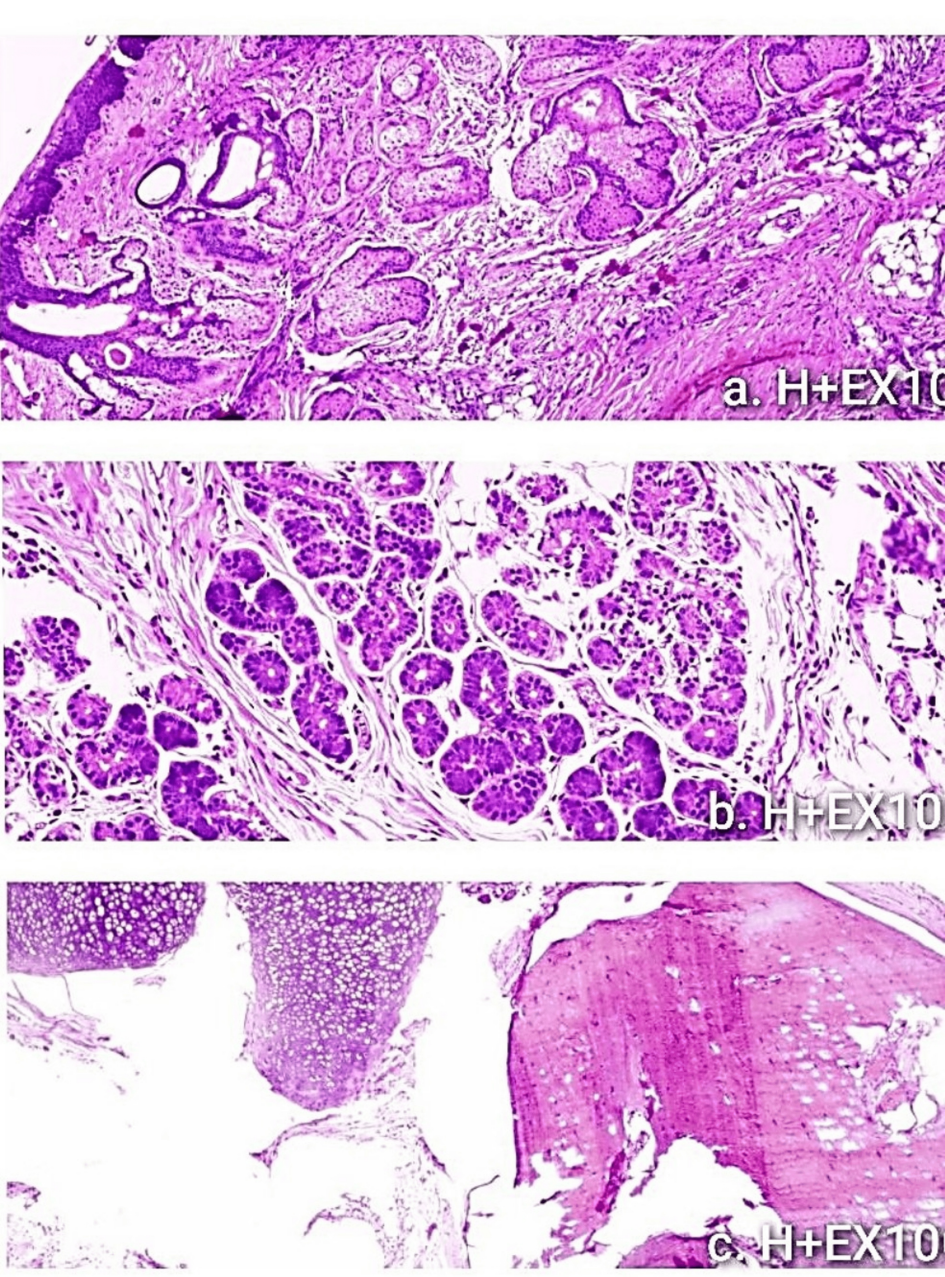
Teratoma composed of mature tissue representing three embryonic layers. a) Skin with sebaceous glands (ectodermal), b) serous glands (endodermal), and c) cartilage and bone (mesodermal)

With those clinical and laboratory findings, the surgeon on call decided to proceed to immediate operation; however, an abdominal ultrasound with full bladder (vaginal scan was excluded), one hour after initial examination and just before entering the theatre, revealed a right-side ovarian cyst measuring 6 cm, with features of a dermoid cyst (unilocular cyst with hyperechogenic protuberance and fat fluid appearance) (Figure 1). Considering the new sonographic evidence, cancer indices were additionally requested preoperatively as expected by the protocol. This finding did not alter the initial surgeon’s decision, and in the operating room, a McBurney’s incision was performed. The patient underwent an appendectomy at first since the appendix looked inflamed. A meticulous further examination also revealed a large adnexal mass, and the gynecologist was called. The initial incision was expanded by one centimeter, and an enlarged edematous gangrenous right adnexa with torsion was extracted (Figure 2). The twisted adnexa was released, set to its anatomical position, and expectant management for ovarian preservation was considered, although necrosis was obviously present. Unfortunately, the necrotic appearance did not improve, and no blood supply was re-established to the ovary. Thus, right salpingoophorectomy was performed (Figure 3). The uterus and the left adnexa were also examined and were found to be normal. The histopathological examination of the specimen showed an ovarian teratoma composed of mature tissue representing three embryonic layers with no malignant cells (Figure 4). The appendix was also inflammatory but the pathologist attributed the inflammation to the ovarian torsion. From the family history, no familial ovarian teratoma was recorded. Laboratory examination on admission, during hospitalization, and after one year and again at two years follow-up was recorded (Table 1). On the last visit, menarche was not present yet. Abdominal ultrasound in the first year showed a normal uterus and left ovary without abnormal pathology, while in the last ultrasound, small follicles were present.

## Discussion

AT is a gynecological emergency presenting usually at reproductive age. Approximately 2.7%-3% of all cases with acute pelvic pain in females are caused by ovarian torsion. AT accounts for 20%-30% of all ovarian operations in the pediatric population between nine and 14 years old, and in these patients, timely ovarian detorsion is considered crucial so as to prevent damage to the organ [3,7]. The presence of ODCs is a common cause of adnexal torsion, reaching a prevalence of 5%-21% women, but it could reach even 22% if the younger population, including premenarchal girls and adolescents, are also added [1]. ODCs are mostly unilateral, but they could be bilateral in approximately 10% of the cases [2,8].

The size of the cyst is considered one of the major causes of torsion. In general, relatively small cysts with a diameter of less than 50 mm and large cysts of more than 100 mm (their size restricts their movements) are considered less likely to cause torsion, while cysts with a diameter between 60 and 90 mm are of greater risk. Other risk factors include tubal spasm, elongated fallopian tubes, and ligaments with increased movement. The role of enhanced hormonal activity in neonates and in premenarchal girls is still under consideration [1,3].

The clinical manifestation of AT is often obscure, especially in cases where no adnexal mass is present [5,9]. In general, pain, nausea, and vomiting are the main presenting symptoms. Laboratory examination could reveal increased leukocytes and CRP, which are associated with ischemia [4]. Additionally, the examination of tumor markers, such as serum human chorionic gonadotropin (β-HCG), α-fetoprotein (αFP), carcinoembryonic antigen (CEA), and cancer antigen 125 (CA-125), could be used to detect the type of tumor in general. More specifically, β-hCG and αFP are suggestive of trophoblastic tumors, and CEA and CA-125 could be elevated in benign and malignant tumors; however, some authors noticed increased markers in children who experienced ovarian torsion on otherwise normal ovaries [3,10-12].

Diagnostic imaging studies include ultrasound, computed tomography (CT), and magnetic resonance imaging (MRI), increasing the sensitivity and specificity of detecting ovarian teratomas. A meta-analysis showed that ultrasound has a sensitivity of 0.79 and a specificity of 0.76 compared to MRI at 0.81 and 0.91, respectively [13].

However, in urgent cases, CT and MRI may delay surgical intervention. Ultrasound by an experienced sonographer is reported to have higher diagnostic accuracy than CT (79% compared to 42%, respectively) for adnexal torsion without exposure to radiation and is considered overall the most used and accurate method for diagnosis [10]. In the pediatric population, the examination of the ovaries could be performed only trans-abdominally with the probe set above the symphysis pubis. A full bladder is useful in creating an acoustic window and providing a better view of the ovaries [4]. Specific features are considered helpful for the evaluation of the adnexa in cases of suspected torsion. An ovarian diameter of more than 4 cm accompanied by ovarian asymmetry and enlargement is considered the most sensitive marker of torsion. A mean ovarian diameter of 5 cm has 91% sensitivity and 92% specificity for suspected torsion [14,15].

Other sonographic findings include increased echogenicity and heterogeneity due to venous congestion and edema, peripheral follicles present due to stromal edema (follicular ring sign), medialization of the ovary accompanied by displacement of the uterus towards the affected ovary, and the double bladder sign in cases where the torsion is caused by a large ovarian cyst [3,4,14].

Color flow Doppler could be useful in determining the reduction or absence of the blood flow and, if possible, in localizing the site of torsion. It has to be noted though that, in ovarian torsion, arterial flow is present in 1/3 of the cases since the ovary has a dual blood supply [4]. Prieto et al. reported that only 38% of twisted ovaries were found to have a lack of Doppler flow [6]. Stark et al. observed that less than 50% of confirmed adnexal torsion at surgery had decreased or absent flow [16], while Spinelli et al. in their retrospective study stated that the blood supply was decreased or absent in 64% of the patients [3].

The differential diagnosis of AT includes acute appendicitis, gastroenteritis, renal colic in premenarchal girls, ectopic pregnancy, and pelvic inflammatory disease in the rest of the female population [3]. In the literature, a unique case of a twisted ovarian dermoid cyst with concomitant acute appendicitis is reported [17].

In general, surgical operation of a diagnosed large dermoid cyst is inevitable due to the higher risk of torsion, spontaneous rupture, or malignancy. Ovarian torsion is an emergency situation, and the first approach should be detorsion of the twisted ovary, followed by adnexal sparing surgery with isolated resection of the cyst and ovarian preservation. If this is not feasible, adnexectomy must be carried out. Surgical approach could be performed by traditional laparotomy or laparoscopy. However, in the pediatric population, the preferred method between the two techniques remains controversial. Laparotomy could be considered in large bilateral cysts or when suspicious pre-surgical findings of malignancy exist. Minimally invasive technique is also considered a safe and effective method of managing ovarian dermoid cysts [2]. Spinelli et al. reported that laparoscopy was statistically correlated with conservative treatment, while open approach or conversion from laparoscopy to laparotomy was associated with more oophorectomies [3]. Furthermore, in the laparoscopic approach, the main concern should be to avoid cystic rupture and spillage of the content in the abdominal cavity, especially when only cystectomy is performed. In cases of expected cystic rupture, adhesion formation and chemical peritonitis could be minimized by the use of a laparoscopic bag and meticulous saline lavage. Meticulous technique should also be performed during the laparoscopic cystectomy in order to achieve complete resection and reduce the recurrence rate [2,3,18]. In those cases, surgeons' experience is of major importance.

## Conclusions

In the presence of ovarian dermoid cysts, AT is a common complication, especially in young age,s and is considered a surgical emergency. In our case, the initial diagnosis and the surgical approach with McBurney’s incision did not alter the final result. The removed appendix was inflammatory due to ovarian necrosis, and the revealed adnexa appeared already gangrenous, leading to inevitable ovarian loss. This report highlights the critical need for vigilance in these premenstrual patients with undiagnosed dermoid cysts, as no universal screening exists, making pediatrician awareness, early detection by ultrasound, and prompt surgery vital for ovarian preservation. The neighboring organs, such as the appendix, should also be examined and removed if inflammation is also present.
